# Differentiation of *Campylobacter jejuni* and *Campylobacter coli* Using Multiplex-PCR and High Resolution Melt Curve Analysis

**DOI:** 10.1371/journal.pone.0138808

**Published:** 2015-09-22

**Authors:** Banya Banowary, Van Tuan Dang, Subir Sarker, Joanne H. Connolly, Jeremy Chenu, Peter Groves, Michelle Ayton, Shane Raidal, Aruna Devi, Thiru Vanniasinkam, Seyed A. Ghorashi

**Affiliations:** 1 School of Animal and Veterinary Sciences, Charles Sturt University, Wagga Wagga, NSW, Australia; 2 Graham Centre for Agricultural Innovation (NSW Department of Primary Industries and Charles Sturt University), Wagga Wagga, NSW, Australia; 3 Birling Avian Laboratories, Bringelly, NSW, Australia; 4 The University of Sydney, Sydney, NSW, Australia; 5 School of Biomedical Sciences, Charles Sturt University, Wagga Wagga, NSW, Australia; Naval Research Laboratory, UNITED STATES

## Abstract

*Campylobacter* spp. are important causes of bacterial gastroenteritis in humans in developed countries. Among *Campylobacter* spp. *Campylobacter jejuni* (*C*. *jejuni*) and *C*. *coli* are the most common causes of human infection. In this study, a multiplex PCR (mPCR) and high resolution melt (HRM) curve analysis were optimized for simultaneous detection and differentiation of *C*. *jejuni and C*. *coli* isolates. A segment of the hippuricase gene (*hipO*) of *C*. *jejuni* and putative aspartokinase (*asp*) gene of *C*. *coli* were amplified from 26 *Campylobacter* isolates and amplicons were subjected to HRM curve analysis. The mPCR-HRM was able to differentiate between *C*. *jejuni and C*. *coli* species. All DNA amplicons generated by mPCR were sequenced. Analysis of the nucleotide sequences from each isolate revealed that the HRM curves were correlated with the nucleotide sequences of the amplicons. Minor variation in melting point temperatures of *C*. *coli* or *C*. *jejuni* isolates was also observed and enabled some intraspecies differentiation between *C*. *coli* and/or *C*. *jejuni* isolates. The potential of PCR-HRM curve analysis for the detection and speciation of *Campylobacter* in additional human clinical specimens and chicken swab samples was also confirmed. The sensitivity and specificity of the test were found to be 100% and 92%, respectively. The results indicated that mPCR followed by HRM curve analysis provides a rapid (8 hours) technique for differentiation between *C*. *jejuni* and *C*. *coli* isolates.

## Introduction

Thermophilic *Campylobacter*s, *C*. *jejuni* and *C*. *coli*, are the leading causes of human foodborne bacterial gastroenteritis worldwide, and are of major public health significance [1].*Campylobacter* has been identified as the major source of food poisoning in the United States [2], Europe [1, 3] and Australia [4].

The prevalence of *Campylobacter* has been studied in a number of farm animals including cattle, sheep, pigs, and chickens [5, 6]. However, exposure to contaminated food of poultry origin has been considered to be the main risk factor for *Campylobacter* infection in humans [4, 7, 8].

The routine testing of foodborne pathogens, such as *Campylobacter* spp., in animal production is an essential component of integrated food safety management systems [9]. Surveillance data, describing the on-farm prevalence and contamination levels of *Campylobacter* spp. in the slaughterhouse, can be used for the implementation of food safety policies and the development and evaluation of intervention strategies to eliminate or mitigate the risk to the consumer. With innovation being the driving force, there is a constant need to improve diagnostic techniques that can rapidly and accurately detect and identify the foodborne pathogens such as *Campylobacter*. Molecular techniques with high sensitivity and specificity are now considered a gold standard test for some pathogens [10, 11], however, it is important to understand the limitations of these techniques in the detection of enteric pathogens from faecal samples [12].

Compared to classical phenotypic techniques for the subtyping of *Campylobacter* spp., genotyping methods are rapid, cost-effective and have been proven to be useful in epidemiological investigations [13, 14]. Various genotyping techniques have been used for differentiation of *Campylobacter* spp. [15]. Pulsed-field gel electrophoresis (PFGE) has a high discriminatory power and has been extensively used as a gold standard method [16, 17]. However, it is labour-intensive and difficult to standardise between different laboratories [18, 19]. Other molecular methods have been used such as Multilocus Sequence Typing (MLST) [20], triplex Polymerase Chain Reaction (PCR)[21], PCR and restriction fragment length polymorphism (RFLP)[22, 23], real-time PCR [24, 25], multiplex PCR (mPCR) [26–28], ribotyping, flagellin (fla) typing [29], and amplified fragment length polymorphism (AFLP) [30]. The *flaA* gene is a common feature of *C*. *jejuni* and *C*. *coli* and has been widely used for genotyping of the species using PCR followed by RFLP and short variable region (SVR) sequencing [29, 31]. Despite extensive use of *flaA*-based typing techniques, this method may not be reliable since *flaA* alleles are unstable due to recombination [32] and intra-species exogenous DNA uptake [33].

It has been established that co-colonisation of host animals with more than one bacterial species can occur [34, 35] and this has also been observed in human clinical cases [36]. Therefore, molecular tests such as mPCR that have the potential to simultaneously detect multiple genotypes would be valuable in these circumstances. The development of fluorescent DNA binding dyes with enhanced saturation properties has permitted a more accurate evaluation of nucleotide sequence variation based on the analysis of DNA melting curves. The technique used in this study, which is referred to as high resolution melt (HRM) curve analysis, has been used for genotyping of *C*. *jejuni* [37, 38], and later *C*. *jejuni and C*. *coli* [39]. However, in previous studies, the detection and differentiation between *Campylobacter* species was made based on visual interpretation of differences in melt curves.

The aim of the current study was to optimize a mPCR-HRM curve analysis technique using non-subjective interpretation of the data derived from HRM curve analysis and to evaluate its discriminatory power for the differentiation of *C*. *jejuni*, *C*. *coli* and intraspecies without requiring enrichment prior testing.

## Materials and Methods

### *Campylobacter* Strains

Twenty-six *Campylobacter* isolates were tested in this study and are shown in Table 1. *Campylobacter* ATCC29428 and ATCC33559 strains were used as controls for *C*. *jejuni* and *C*. *coli*, respectively. All *C*. *jejuni and C*. *coli* isolates were provided by Birling Avian Laboratories, New South Wales, Australia. The nine *C*. *jejuni* isolates of chicken droppings were previously tested by different techniques [18, 39]. *Campylobacter* isolates of broiler chicken carcass origin were cultured by Birling Avian Laboratories using standard microbiological methods. All isolates were sub-cultured on sheep blood agar (ThermoFisher Scientific, Australia) and incubated at 42°C under microaerophilc conditions (83% N_2_, 4% H_2_, 8% O_2_ and 5% CO_2_). After 72 hours, all cultured plates were observed for purity. The suspected *Campylobacter* colonies were confirmed by phase contrast microscopy for characteristic corkscrew-like motility and spiral shaped cells. A single representative colony from each culture was used for DNA extraction. Pure cultures of the isolates were stored at -70°C using a cryovial (Microbank^TM^, Pro-Lab Diagnostics, Australia) for further use. In addition, clinical samples including nine human faecal samples previously confirmed positive for *Campylobacter* were collected from Westmead Hospital (Sydney, Australia) and 25 swab samples from chicken carcases were used to evaluate the developed PCR-HRM technique for its potential to differentiate *C*. *jejuni* and *C*. *coli*.

**Table 1:** Identification, species, source and mean±SD of the melting points and GCP for *C*. *jejuni and C*. *coli* isolates when using ATCC29428 and ATCC33559 as reference strains, respectively.

Isolate ID.	*flA* types ^a^	Species	Source	No. of times tested	Peak 1 (°C)	Peak 2 (°C)	GCP±SD (%)	GenBank Acc. No.
C669	NT^b^	*C*. *coli*	Chicken dropping	17	79.2 ±0.3	82.9±0.3	63.5±6.5	KF830146
C1280	NT	*C*. *coli*	Chicken dropping	19	80.0±0.4	83.3±0.3	74.5±3.8	KF830147
C326	NT	*C*. *coli*	Chicken dropping	22	79.4±0.4	83.5±0.3	96.2±2.0	KF830145
C286	NT	*C*. *coli*	Chicken dropping	19	79.2±0.4	83.3±0.4	90.5±3.4	KF830152
D912	NT	*C*. *coli*	Chicken dropping	19	79.5±0.4	83.6±0.4	94.7±3.2	KF830153
BAL172668	NT	*C*. *coli*	Broiler chicken carcass	21	79.9±0.4	83.7±0.3	82.4±7.7	KF830150
BAL172832	NT	*C*. *coli*	Broiler chicken carcass	19	79.3±0.4	83.5±0.3	97.3±1.9	KF830151
BAL172104	NT	*C*. *coli*	Broiler chicken carcass	19	79.7±0.4	83.4±0.4	90.6±2.8	KF830149
ATCC33559	NT	*C*. *coli*	Pig feces	27	79.4±0.4	83.5±0.3	99.5±2.7	KF830148
C350	XV	*C*. *jejuni*	Chicken dropping	19	81.2±0.4		65.0±6.0	KF830154
C1270	XXIII	*C*. *jejuni*	Chicken dropping	16	80.8±0.4	82.6±0.4	91.5±6.3	KF830155
C660	XI	*C*. *jejuni*	Chicken dropping	19	81.1±0.4		63.6±8.0	KF830164
L131	VIII	*C*. *jejuni*	Chicken dropping	16	81.1±0.3	83.4±0.3	93.5±1.2	KF830167
M2	I	*C*. *jejuni*	Chicken dropping	16	81.0±0.4		73.9±7.6	KF830168
C358	NT	*C*. *jejuni*	Chicken dropping	16	81.0±0.3		63.8±8.2	KF830163
C1212	V	*C*. *jejuni*	Chicken dropping	16	81.1±0.3		64.1±7.6	KF830165
D190	NT	*C*. *jejuni*	Poultry farm environment	4	80.6±0.4		58.8±1.7	KF830166
N15	LIII (XXIV)	*C*. *jejuni*	Chicken dropping	7	80.5±0.3	82.5±0.4	95.9±0.5	KF830169
N70	XXVI	*C*. *jejuni*	Chicken dropping	7	80.7±0.5		85.3±3.1	KF830170
A529	I	*C*. *jejuni*	Chicken dropping	15	80.6±0.9		88.1±5.4	KF830162
BAL172630	NT	*C*. *jejuni*	Broiler chicken carcass	13	80.7±0.5		75.8±8.6	KF830161
BAL172236	NT	*C*. *jejuni*	Broiler chicken carcass	19	81.0±0.3	83.3±0.4	83.3±11.6	KF830159
BAL172643	NT	*C*. *jejuni*	Broiler chicken carcass	9	80.7±0.4	82.5±0.3	92.9±4.9	KF830160
BAL172084	NT	*C*. *jejuni*	Broiler chicken carcass	12	80.8±0.4		76.9±6.0	KF830158
ATCC29428	NT	*C*. *jejuni*	Human feces	27	80.6±1.2	83.0±0.5	98.2±0.7	KF830157
NCTC11351	NT	*C*. *jejuni*	Bovine feces	21	81.1±0.4		72.5±4.1	KF830156

^a^ *flA* types were determined by Merchant-Patel, 2008

^b^ Not tested

### Ethics Statement

Human faecal samples from unidentifiable patients were provided for research purposes by Westmead hospital (Sydney, Australia). The study was reviewed and approved by Human Research Ethics Committee (HREC) at Charles Sturt University (permit No. 2012/125). The opportunistic chicken swab samples were collected from chicken carcases in abattoir (Poultry Processing Plant, NSW, Australia) where chickens were slathered for meat consumption under the Food Act 2003 (NSW) and Food Regulation 2010. Collected samples from chicken carcase were also used for research study.

### DNA Extraction

Total genomic DNA was extracted from *Campylobacter* cultures using Wizard® SV Genomic DNA Purification kit (Promega, cat no. A2360, VIC, Australia) according to the manufacturer’s instructions. Briefly, 0.5 ml of *Campylobacter* culture was pelleted by centrifugation at 14, 000 x *g* for 2 min. The pellet was resuspended in lysis/RNase solution and incubated at 80°C for 10 min. The bacterial cell lysate was transferred into the Wizard® SV mini-column assembly and centrifuged at 13,000 × *g* for 3 min. The column was washed with 650 μl of wash buffer three times and each time was subjected to centrifugation at 13, 000 *g* for 2 min. The DNA was eluted from the matrix using 50 μl distilled PCR-grade water. The extraction of DNA from clinical faecal samples and chicken carcase swab samples was performed using QIAmp DNA stool Mini Kit (Qiagen, Australia) and Wizard® SV Genomic DNA Purification kit (Promega, cat no. A2360, VIC, Australia) according to the manufacturer’s instructions. All extracted DNA were quantified using the Nanodrop2000 (ThermoFisher Scientific, Australia) and the concentration of each DNA sample adjusted to 5 ng/μl for subsequent mPCR amplification or stored at -20°C for future use.

### mPCR Amplification

The N-benzoylglycine amidohydrolase or hippuricase (*hipO*) and putative aspartokinase (*asp*) genes were selected for identification of the species *Campylobacter jejuni* and *coli*, respectively. The primer sequences for the gene targets were selected from published literature [40–43]. The mPCR was optimized using two primer sets (HIP400F) 5’-GAAGAGGGTTTGGGTGGTG-3’ and (HIP1134R) 5’-AGCTAGCTTCGCATAATAACTTG-3’ and (CC18F) 5’-GGTATGATTTCTACAAAGCGA-3’ and (CC519R) 5’-ATAAAAGACTATCGTCGCGTG-3’ for amplification of 735 and 500 bp fragments of *C*. *jejuni* and *C*. *coli*, respectively. BLAST results of primer sequences showed specific identity to *C*. *jejuni* and *C*. *coli*.

The mPCR amplification was performed in 25 μl reaction volumes on an I-Cycler (Bio-Rad Laboratories Pty., Ltd. Gladesville, Australia). The reaction mixture contained 1 μl extracted genomic DNA, 25 μM of each primer, 1.5 mM MgCl_2_, 1250 μM of each dNTP, 5 μM SYTO^®^ 9 green fluorescent nucleic acid stain (Life Technologies Australia Pty Ltd., Mulgrave, Australia), 1× GoTaq^®^ Green Flexi Reaction Buffer (Promega) and 1 U of Go Taq DNA polymerase (Promega Corporation, USA). The optimal mPCR conditions were initial denaturation at 96°C for 3 min, then 35 cycles of 96°C for 30 s, 55°C for 30 s and 72°C for 30 s, and a final extension of 72°C for 5 min.

### Sequencing and Nucleotide Sequence Analysis of mPCR Amplicons

The mPCR amplicons of all tested samples were purified using the QIAquick^®^ PCR Purification Kit (Qiagen, cat no. 28104, Chadstone, Australia) following the manufacturer’s instructions. Purified amplicons were subjected to automated sequencing (BigDye^®^ Terminator v3.1, Applied Biosystems, Life Technologies Australia Pty Ltd., Mulgrave, Australia) in both directions, using the same primers for each species as used for mPCR. The nucleotide sequences were analysed using ClustalW [44] and BioEdit Sequence Alignment Editor (version 6.0.9.0).

### High-Resolution Melt Curve Analysis

HRM curve analysis was performed in a Rotor-Gene™ 6000 thermal cycler (Qiagen, Chadstone, Australia). The mPCR products were subjected to 0.5°C/s ramping between 70°C and 90°C. All specimens were tested in triplicates and their melting profiles were analysed using Rotor Gene 1.7.27 software and the HRM algorithm provided. The normalisation regions of 77–78°C and 85–86°C were used for analysis of melt curves. A reference strain for each target gene (*hipO* and *asp* gene) was set as ‘genotype’ (ATCC29428 for *C*. *jejuni* and ATCC33559 for *C*. *coli*) and the average HRM genotype confidence percentage (GCP) (the value attributed to each strain being compared to the genotype with a value of 100% indicating an exact match) for the replicates was predicted by the software.

The GCPs of all *C*. *jejuni* and all *C*. *coli* specimens were averaged separately and the standard deviation (SD) calculated for each and used to establish the GCP range for *C*. *jejuni* and *C*. *coli* strains cut off point. The values above and below cut off points were then used for identification of *Campylobacter* species.

To evaluate the intraspecies differentiation power of mPCR-HRM and to detect minor differences between *C*. *jejuni* or *C*. *coli* isolates, the mean GCPs±SD of ATCC29428 and ATCC33559 were calculated and used as cut off value to evaluate the differences between isolates within each *Campylobacter* species.

## Results

Amplified mPCR products from different *Campylobacter* isolates were analysed by gel electrophoresis (S1 Fig). Each *C*. *jejuni* and *C*. *coli* isolate generated only one single amplicon approximately 735 and 500 bp, respectively and non-specific amplification was not observed. The primers used in this study were not able to amplify DNA fragments when the test was performed on (*Campylobacter* negative) stool samples.

The sensitivity of the mPCR-HRM in detecting *Campylobacter* species was determined by testing serial 10 fold dilutions of DNA from reference strains (ATCC33559 and ATCC29428). The first PCR dilution received 2 ng DNA template. Results showed that the sensitivity of the test was 2x10^-5^ and 2x10^-4^ ng DNA for *C*. *jejuni* and *C*. *coli* respectively.

The sensitivity and specificity of the test for differentiation of *Campylobacter* species in clinical samples were also determined using the receiver operating characteristic (ROC) analysis at three different cut off points (≥10, ≥60 and ≥90). The sequencing results were used as gold standard. The sensitivity and specificity of the test were 100% and 92% respectively, when cut off point was set ≥60 and were superior compared to those of cut off points ≥10 and ≥90.

### Differentiation of *C*. *jejuni* and *C*. *coli* Strains by Conventional and Normalised HRM Curve Analysis

**Fig 1:**
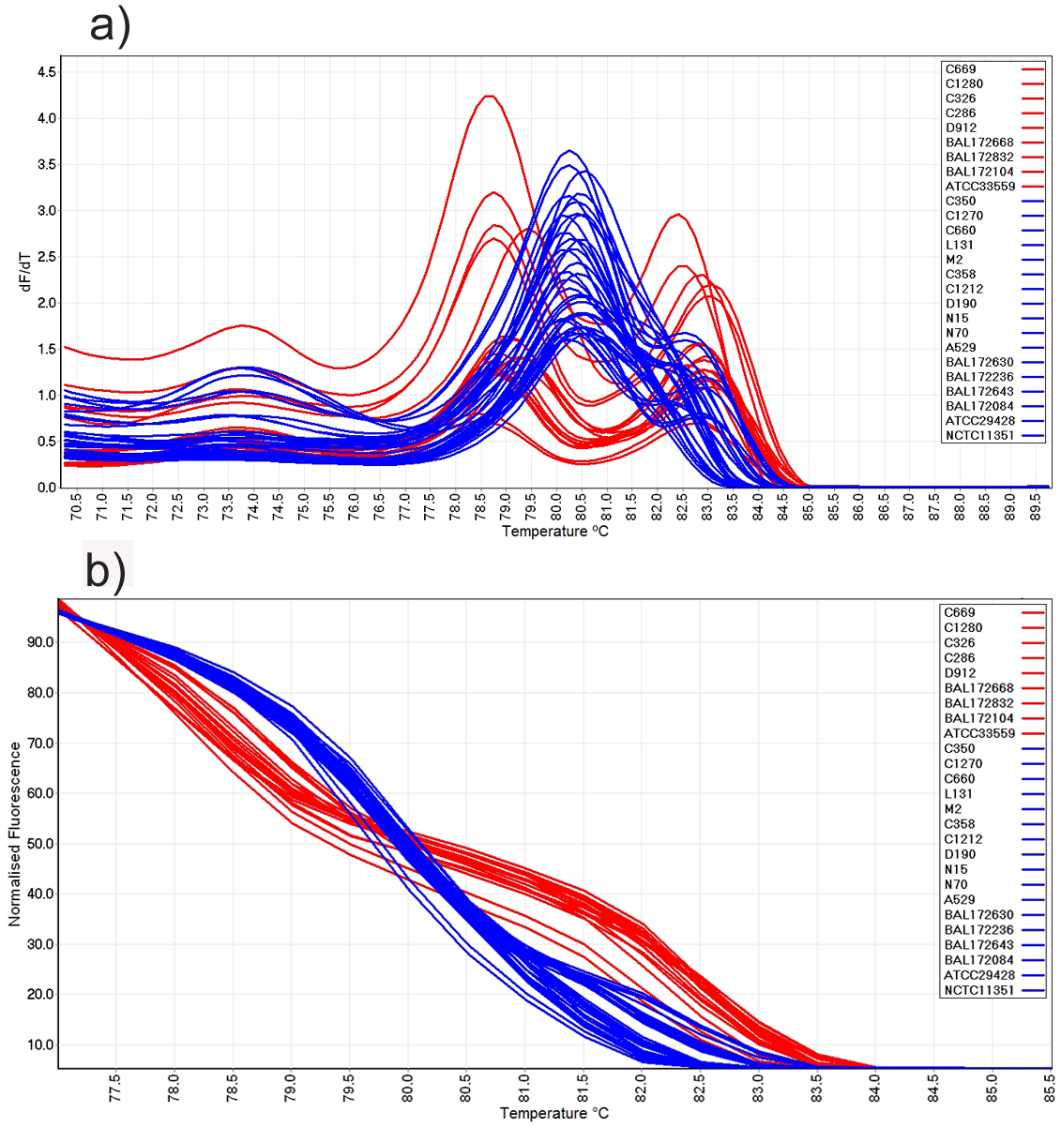
Conventional and normalized melt curve analysis of *Campylobacter* strains. (a) Conventional and (b) normalized HRM curve analysis of mPCR amplicons for *C*. *jejuni* (blue colour) and *C*. *coli* (red colour) isolates.

The mPCR amplicons from 26 *Campylobacter* isolates were subjected to HRM curve analysis (Fig 1). Visual examination of the conventional melt curves at different ramp temperatures revealed that 0.5°C/s resulted in most strains showing distinct profiles. Overall, two major conventional and normalized melt curve profiles were detected (Fig 1a and 1b).

**Fig 2:**
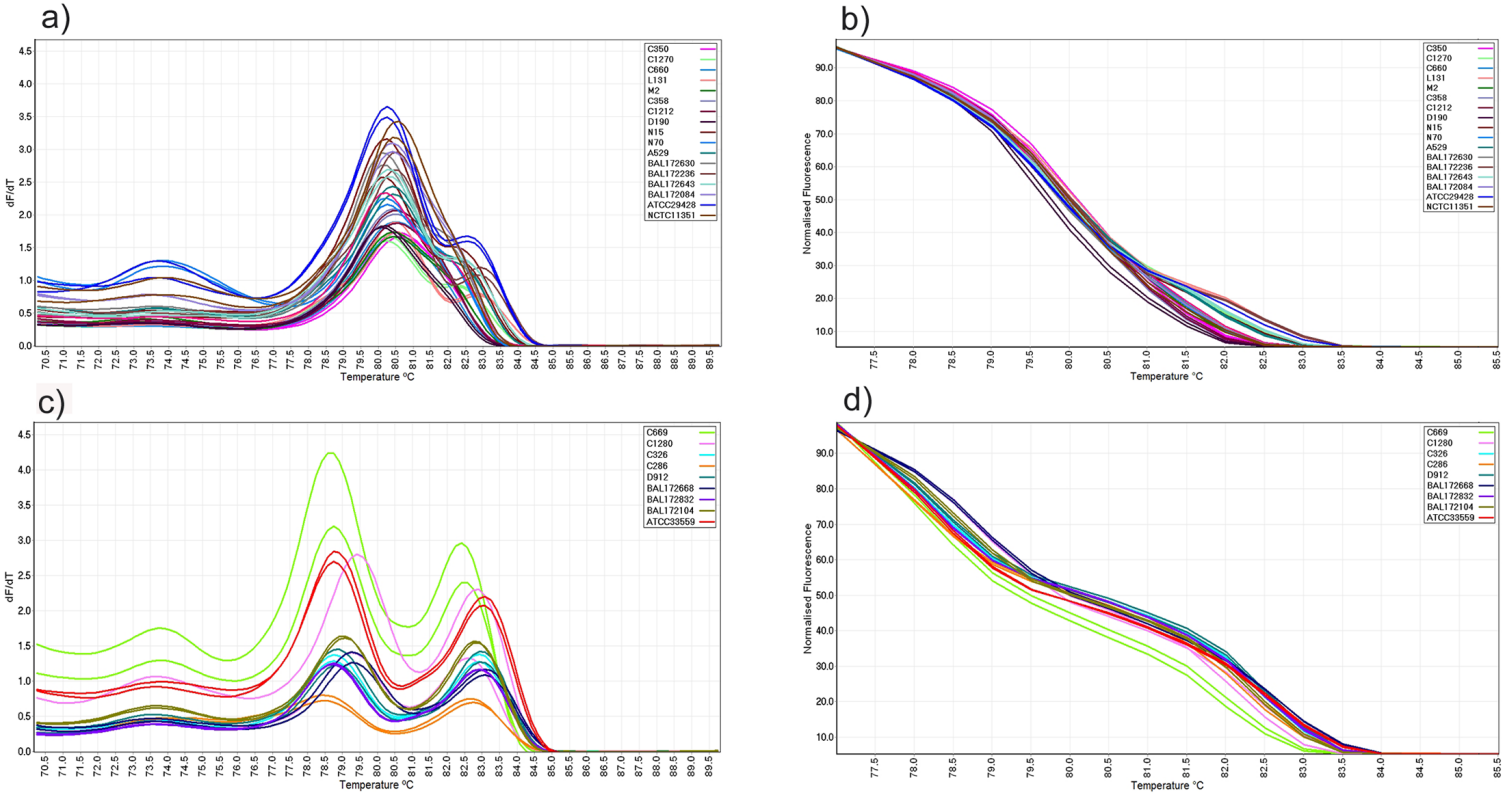
Conventional and normalized melt curve analysis of *Campylobacter* species. (a) Conventional and (b) normalized melt curve analysis of mPCR amplicons from *C*. *jejuni* isolates. All isolates produced a single peak while 9 isolates generated an additional shoulder peak at higher temperatures. (c) Conventional and (d) normalized melt curve analysis of mPCR amplicons from *C*. *coli* isolates. All isolates produced 2 peaks in conventional melt curves.

All *C*. *jejuni* isolates generated one peak in the range of 80.5°C–83.4°C. However, nine out of 17 *C*. *jejuni* isolates (C1270, L131, N15, N70, A529, BAL172630, BAL172236, BAL172643 and ATCC29428) also generated a shoulder peak at a higher temperature. Therefore, among *C*. *jejuni* strains two distinct conventional and normalized melting patterns could also be identified (Fig 2a and 2b). All nine *C*. *coli* strains produced two peaks in the range between 79.2°C–83.7°C in the conventional curve (Fig 2c).

*Campylobacter coli* specimens, C326 and D912, both produced two peaks, one peak at 78.75°C and one at 82.90°C and had similar conventional and normalized curves (Fig 2d). The C669 *C*. *coli* isolate produced two peaks, one peak at 78.75°C and one at 82.5°C and generated conventional and normalized melt curves which were different from the rest of the *C*. *coli* isolates.

### Non-Subjective Differentiation of *Campylobacter* Species Using GCPs

High resolution melt curve analysis for mPCR amplicons using templates from DNA extractions and mPCRs run on different days showed slight shifts in melting temperatures, but the shape and the relative position of the conventional and normalized melt curves remained unchanged.

The mean and SD of melting points for the nine *C*. *coli* and the 17 *C*. *jejuni* isolates and the mean GCP resulting from different runs of mPCR-HRM curve analysis are shown in Table 1.

Using GCPs of all *C*. *coli* isolates, a cut-off value was generated as a mathematical model to assess the relationship of the isolates without visual interpretation by the operator. The mean GCP of *C*. *coli* specimens was 87.4 and the mean SD was 12.8. The value of 2SD was subtracted from the average GCP to determine a cut off point. A cut off point value of 61.8 was determined for *C*. *coli* genotypes. Thus the GCP range of the *C*. *coli* isolates was determined to be 61.8–100 and was used for detection of all *Campylobacter* isolates.

Similarly, GCPs of all *C*. *jejuni* isolates were used to determine the cut off point for *C*. *jejuni* isolates. The mean GCP of *C*. *jejuni* genotypes was 78.9 and the mean SD was 12.6. The value of 2SD was subtracted from the average GCP to determine the cut off point for *C*. *jejuni*. A cut off point value of 53.7 was determined for *C*. *jejuni* genotypes. Thus the GCP ranges for the *C*. *jejuni* samples were determined to be 53.7–100 and this cut-off value was used for detection of all *Campylobacter* isolates.

To assess the discriminatory power of the mPCR-HRM technique (i.e. the ability of the method to differentiate between *C*. *jejuni* and *C*. *coli* isolates) ATCC29428 and ATCC33559 were used as reference strains for *C*. *jejuni and C*. *coli*, respectively, and cut off values were applied for each species.

When ATCC33559 was used as reference genotype with a cut off value of 61.8, all *C*. *coli* isolates produced a GCP ≥61.8 and genotyped as *C*. *coli*. All *C*. *jejuni* isolates also generated GCPs between 6–24 which were <61.8, and therefore were automatically genotyped as ‘variation’.

**Fig 3:**
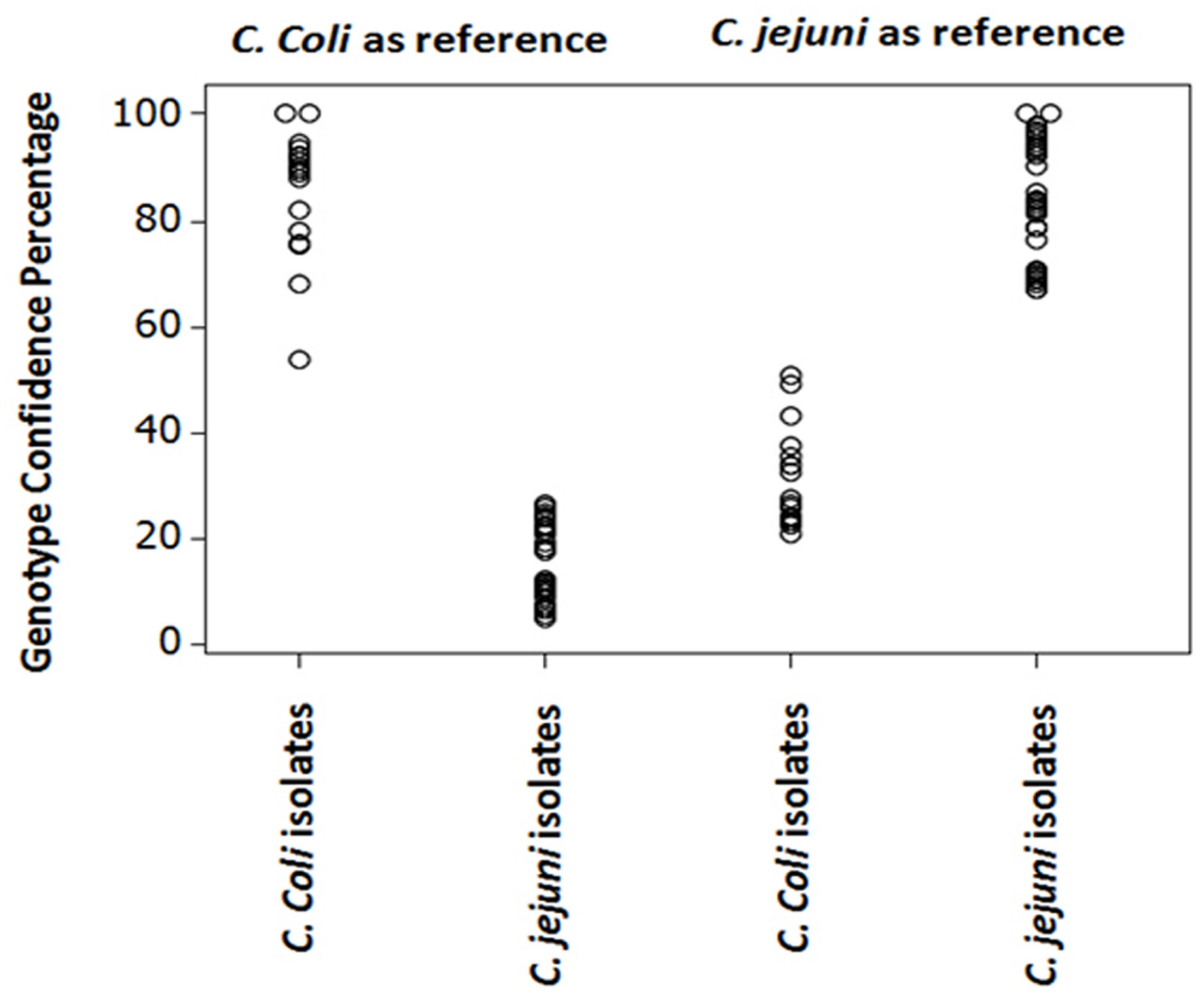
Comparison of the distribution of GCPs from *C*. *jejuni* and *C*. *coli* isolates by individual value plot when ATCC29428 and ATCC33559 were used as reference genotypes, respectively.

When ATCC29428 was used as reference genotype using a cut off point of 53.7, all *C*. *jejuni* isolates were genotyped as *C*. *jejuni* with a GCP ≥53.7 and all *C*. *coli* isolates produced GCPs between 19–48 which were <53.7, and therefore automatically identified as variation. Thus gap between the highest and lowest GCP between *C*. *coli* and *C*. *jejuni* isolates were 27 and 15 when ATCC33559 or ATCC29428 were used as reference genotypes (Fig 3).

### Evaluation of Discriminatory Power of mPCR-HRM in Differentiation of Intraspecies within *C*. *jejuni* and *C*. *coli* Isolates

To assess the differentiation power of newly developed mPCR-HRM technique in detection of minor differences among *C*. *jejuni* or *C*. *coli* isolates, the mean GCPs±SD of *C*. *jejuni* and *C*. *coli* reference strains (ATCC29428 and ATCC33559, respectively) were calculated. The GCP±SD for ATCC29428 and ATCC33559 were 98.2±0.7 and 99.5±2.7, respectively. The value of 2SD was subtracted from the mean GCP and the cut off points of 96.8 and 94.1 were calculated for ATCC29428 and ATCC33559, respectively.

When ATCC33559 was used as reference genotype with a cut off point of 94.1, all *C*. *coli* isolates produced a GCP≤94.1 and genotyped as variation (Table 2). When ATCC29428 was used as reference genotype with a cut off point of 96.8, all *C*. *jejuni* isolates produced a GCP≤96.8 and therefore were automatically genotyped as variation. The only exception was BAL172643 isolate that produced a GCP of 97.9 and could not be differentiated from ATCC29428 by this method (Table 3). However, BAL172643 and ATCC29428 were differentiable by visual examination of their conventional and normalized curves (S2 Fig). The nucleotide sequences of BAL172643 and ATCC29428 were 98.1% identical.

**Table 2:** Intraspecies differentiation (genotypes) within *C*. *coli* isolates when ATCC33559 was used as reference genotype with a cut off point of 94.1

Isolate ID.	Species	GCP±SD (%)	Genotype
C669	*C*. *coli*	58.6±3.8	Variation
C1280	*C*. *coli*	70.7±2.6	Variation
C326	*C*. *coli*	89.8±0.8	Variation
C286	*C*. *coli*	91.4±0.8	Variation
D912	*C*. *coli*	88.5±3.7	Variation
BAL172668	*C*. *coli*	76.5±3.0	Variation
BAL172832	*C*. *coli*	91.4±0.3	Variation
BAL172104	*C*. *coli*	87.3±1.2	Variation
ATCC33559	*C*. *coli*	99.9±0.0	ATCC33559

**Table 3:** Intraspecies differentiation (genotypes) within *C*. *jejuni* isolates when ATCC29428 was used as reference genotype with a cut off point of 96.8.

Isolate ID.	Species	GCP±SD (%)	Genotype
C350	*C*. *jejuni*	67.2±0.8	Variation
C1270	*C*. *jejuni*	93.3±0.2	Variation
C660	*C*. *jejuni*	67.6±0.4	Variation
L131	*C*. *jejuni*	91.4±0.3	Variation
M2	*C*. *jejuni*	77.3±0.6	Variation
C358	*C*. *jejuni*	67.3±0.6	Variation
C1212	*C*. *jejuni*	67.8±1.3	Variation
D190	*C*. *jejuni*	56.5±1.5	Variation
N15	*C*. *jejuni*	95.1±0.5	Variation
N70	*C*. *jejuni*	82.0±1.1	Variation
A529	*C*. *jejuni*	92.1±0.6	Variation
BAL172630	*C*. *jejuni*	82.7±0.6	Variation
BAL172236	*C*. *jejuni*	93.6±0.6	Variation
BAL172643	*C*. *jejuni*	97.9±0.1	ATCC29428
BAL172084	*C*. *jejuni*	80.1±0.2	Variation
ATCC29428	*C*. *jejuni*	99.9±0.1	ATCC29428
NCTC11351	*C*. *jejuni*	76.0±1.5	Variation

### *Campylobacter* HRM Curve Profiles Correlated with Nucleotide Sequence Variation of Tested Specimens

To evaluate the HRM results in detection of similarities or variations within each *Campylobacter* species and whether these differences are correlated with the nucleotide sequences of amplicons, all *C*. *jejuni* and *C*. *coli* amplicons were sequenced and sequence analysis was performed.

The *C*. *jejuni* and *C*. *coli* isolates with distinct HRM curve profiles within each species, showed nucleotide sequence variation to each other consisting of nucleotide deletion, insertion and/or substitution in the *hipO* and *asp* gene, respectively (results not shown).

The highest sequence identity between *C*. *coli* strains was 99.2% between D912 and C326, which produced similar conventional and normalized melt curves (S1 Table). The lowest sequence identity was 93.5% between C669 and BAL172668, which produced two distinct conventional and normalized melt curves.

The highest and lowest sequence identity between *C*. *jejuni* strains was 99.6% between C358 and C1212 and 95.3% between BAL172084 and ATCC29428, respectively (S2 Table). The C358 and C1212 generated similar conventional and normalized melt curves while BAL172084 and ATCC29428 produced two distinct curves in HRM analysis. The correlation of sequence identities and GCP values was calculated to be 0.691 for *C*. *coli* isolates and 0.490 for *C*. *jejuni* isolates using Pearson correlation analysis.

The amplicon size plays a crucial role in HRM curve analysis [45]. Amplicon sizes of 200–400 bp (or less) can increase the detection sensitivity of sequence diversity in tested specimens [46, 47]. However, HRM analysis depends on the number of base pair changes in target DNA segment. If there is large number of nucleotide variations, then larger segments can be targeted. Larger segments of amplicons have been successfully used in PCR-HRM analysis in previous studies [17, 35, 48, 49].

Phylogenetic trees were generated for each *Campylobacter* species based on the sequence alignments (S3 Fig). *Campylobacter* isolates with high sequence identity such as C326 and D912 (*C*. *coli*) or M2 and N70 (*C*. *jejuni*) formed a clade, while isolates with sequence variation formed a sister clade (C1280) or a separate clade (C669) based on the level of sequence diversity.

### Assessment of mPCR-HRM Technique for Its Potential in Detection and Differentiation of *C*. *jejuni* and *C*. *coli* in Clinical Specimens

The mPCR amplicons from faecal samples were subjected to HRM curve analysis and conventional and normalized curves were compared with *C*. *jejuni* and *C*. *coli* reference genotypes. The shape and number of curves and melting point of the peaks were considered in the initial screening. However, genotyping of samples was carried out using Rotor Gene 1.7.27 software. Amplicons of all tested samples were also sequenced for further analysis.

**Fig 4:**
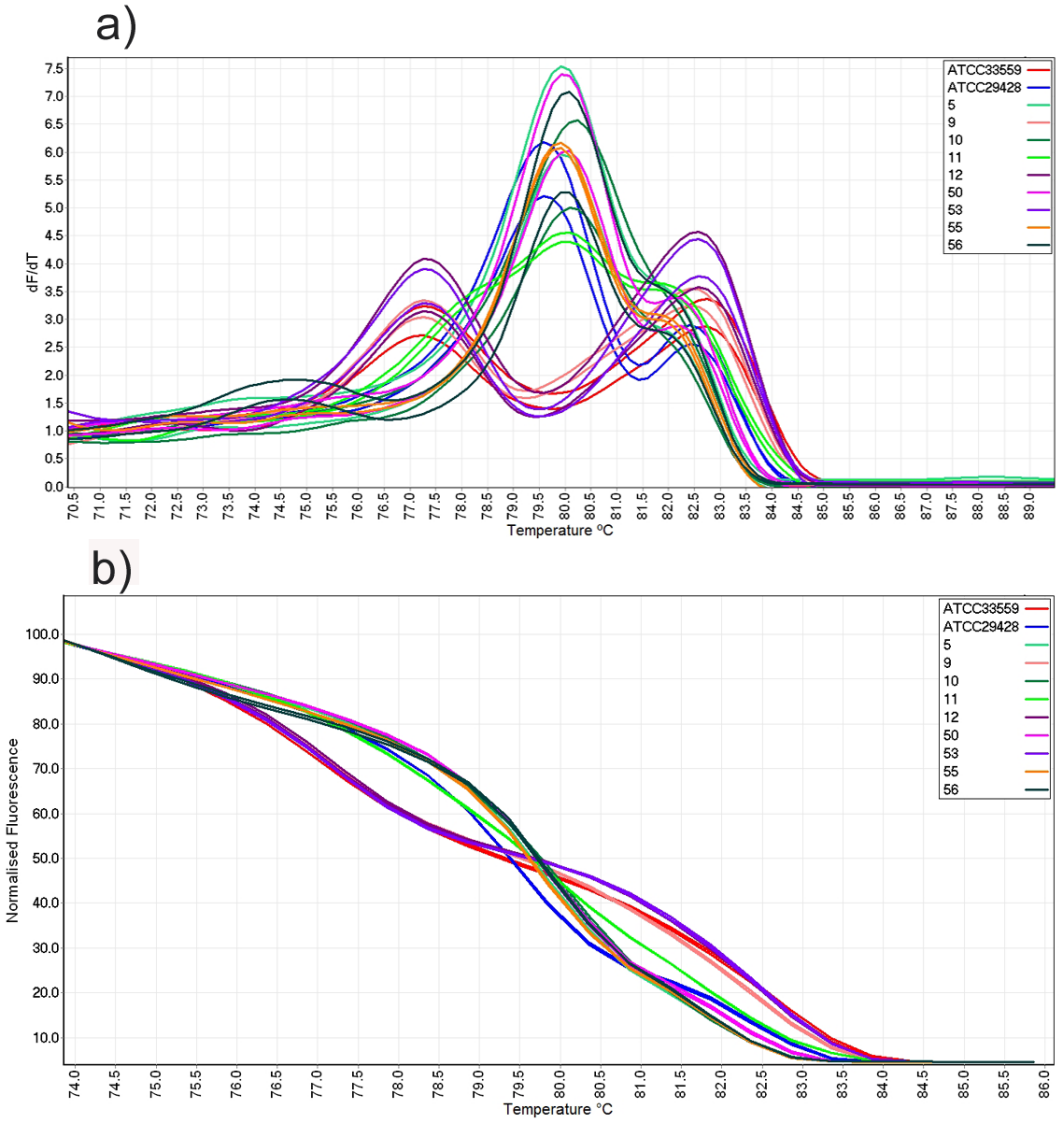
Conventional and normalized melt curve analysis of human clinical samples. (a) Conventional melt curve and (b) normalized HRM curve analysis of mPCR amplicons of human faecal samples.

Examination of conventional HRM curves from human faecal samples revealed that samples 5, 10, 50, 55 and 56 produced a peak between 79.9–80.1°C. Sample 50 also produced a shoulder peak at higher temperature (82.2°C) (Table 4). These curves were comparable with that of ATCC29428 (*C*. *jejuni*) and therefore, were automatically genotyped as *C*. *jejuni* when the cut off point was applied and ATCC29428 was used as reference genotype (Fig 4).

**Table 4:** Clinical human faecal samples tested with mPCR-HRM. Mean±SD of the melting points and GCP produced by isolates when ATCC29428 and ATCC33559 were used as reference strains.

Isolate ID.	Peak 1 (°C)	Peak 2 (°C)	Peak 3 (°C)	GCP±SD (%)	Genotype	GenBank Acc. No.
5	79.9±0.2			80.5±0.5	*C*. *jejuni*	KP164637
9	77.3±0.5	82.6±0.4		96.4±0.4	*C*. *coli*	KP164643
10	80.1±0.8			72.5±1.7	*C*. *jejuni*	KP164638
11	77.8±0.5	80.1±0.3	82.0±0.2	48.2±0.1^a^ 39.0±0.6^b^	Variation	KP164639 and KP164644
12	77.3±0.5	82.6±0.2		85.0±0.3	*C*. *coli*	KP164645
50	80.0±0.1	82.2±0.1		77.4±0.8	*C*. *jejuni*	KP164640
53	77.3±0.5	82.6±0.1		85.2±0.3	*C*. *coli*	KP164646
55	79.9±0.2			81.0±0.8	*C*. *jejuni*	KP164641
56	80.0±0.3			73.5±1.8	*C*. *jejuni*	KP164642
ATCC33559	77.3±0.2	82.6±0.3		99.8±0.1	*C*. *coli*	KF830148
ATCC29428	79.9±0.8	82.4±0.5		99.9±0.0	*C*. *jejuni*	KF830157

^a^, when ATCC29428 was used as reference strain

^b^, when ATCC33559 was used as reference strain

Clinical specimens 9, 12 and 53 each generated a conventional curve with two peaks at 77.3 and 82.6°C which were similar to *C*. *coli* reference strain ATCC33559 (Fig 4a). The normalized curves of these specimens were also similar and all were genotyped as *C*. *coli* when the cut off point was applied and ATCC33559 was used as reference genotype (Fig 4b).

However, specimen 11 produced a conventional curve consisting of one peak at 80°C and two shoulder peaks at 77.8°C and 82.0°C which was different from both reference genotypes (ATCC29428 and ATCC33559) and other clinical specimens (Fig 4a). The normalized curve of sample 11 was also distinct from the rest of samples and therefore, automatically was genotyped as variation when ATCC29428 or ATCC33559 were used as reference genotype (Fig 4b). Results indicated that the test has the capacity to differentiate *C*. *coli* and *C*. *jejuni* in clinical samples without requiring *Campylobacter* isolation in culture or enrichment step prior testing.

Agarose gel analysis of these samples revealed that all specimens identified as *C*. *jejuni* produced a single DNA fragment about 735 bp while all samples identified as C coli generated a single fragment about 500 bp. Sample 11 produced two DNA fragments of 735 and 500 bp similar to *C*. *jejuni* and *C*. *coli*, respectively (data not shown). All amplicons including both DNA fragments of sample 11 were sequenced. All sequences were subjected to a BLAST search (http://blast.ncbi.nlm.nih.gov/Blast.cgi) to determine their identities. Sample 11 contained a mixed infection of *C*. *jejun*i and *C*. *coli*. Results of BLAST search confirmed HRM genotyping. The phylogenetic tree generated based on multiple sequence alignment of clinical samples generated two separate clades for *C*. *jejuni* and *C*. *coli* species (S4 Fig).

**Fig 5:**
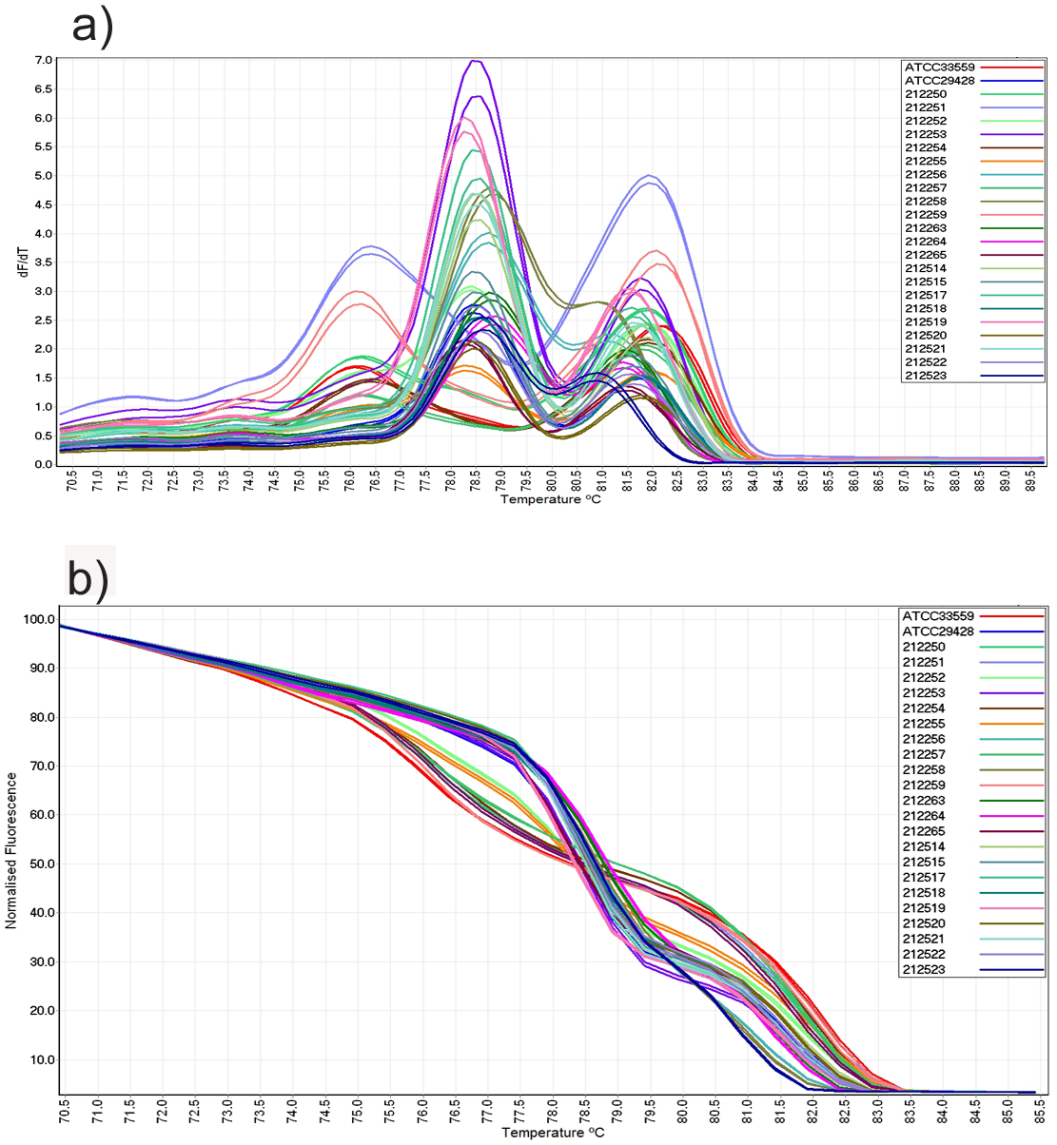
Conventional and normalized melt curve analysis of chicken clinical samples. (a) Conventional melt curve and (b) normalized HRM curve analysis of mPCR amplicons from chicken swab samples.

The mPCR amplicons from 25 chicken carcase swab samples were subjected to HRM curve analysis and were genotyped using Rotor Gene software when cut off points of reference strains were applied (Table 5). Five swab samples were genotyped as *C*. *coli* and generated a peak between 76.60–81.60°C while 15 samples genotyped as *C*. *jejuni* and produced one peak at 79.60–79.65°C or one peak at 78.75–79.65°C and a shoulder peak at higher temperature (81.10–81.40°C) (Fig 5). Three swab samples (212262, 212266 and 212516) did not produce HRM curve or DNA fragment on agarose gel and two samples (212252 and 212255) produced one peak at 79.25°C and two shoulder peak at 76.5°C and 81.25°C which were different to those of *C*. *coli* and *C*. *jejuni* specimens and also produced two DNA fragments of about 735 and 500 bp in agarose gel indicating of mixed infection with the two species.

**Table 5:** Chicken carcase swab samples tested with mPCR-HRM. Mean±SD of the melting points and GCP produced by isolates when ATCC29428 or ATCC33559 were used as reference strains.

	Culture		mPCR-HRM					
			Melting points			Mean GCP±SD (%)		
Isolate ID.	culture	species	Peak 1 (°C)	Peak 2 (°C)	Peak 3 (°C)	ATCC33559 used as reference strain	ATCC29428 used as reference strain	Genotype
ATCC33559	Positive	*C*. *coli*	76.1±0.1	82.1±0.1		99.9±0.1	7.7±0.1	*C*. *coli*
ATCC29428	Positive	*C*. *jejuni*	78.5±0.0	81.8±0.0		7.7±0.3	99.9±0.1	*C*. *jejuni*
212250	Negative	NA^a^	76.2±0.1	81.9±0.3		88.0±0.5	17.5±0.2	*C*. *coli*
212251	Negative	NA	76.4±0.1	81.9±0.2		90.3±0.3	16.3±0.2	*C*. *coli*
212252	Negative	NA	77.5±1.3	80.2±2.4	81.9±0.1	32.4±1.1	49.1±1.1	Variation
212253	Negative	NA	78.5±0.1	81.6±0.1		4.6±0.4	96.3±1.4	*C*. *jejuni*
212254	Negative	NA	76.4±0.0	81.9±0.0		92.2±0.2	11.9±0.2	*C*. *coli*
212255	Negative	NA	76.0±0.1	78.3±0.1	82.0±0.0	49.3±2.1	50.3±2.5	Variation
212256	Negative	NA	78.8±0.1	81.2±0.1		2.5±0.2	71.1±0.9	*C*. *jejuni*
212257	Negative	NA	76.3±0.1	81.8±0.1		81.7±3.2	11.9±0.4	*C*. *coli*
212258	Negative	NA	78.8±0.1	80.9±0.0		1.9±0.2	58.3±2.5	*C*. *jejuni*
212259	Negative	NA	76.2±0.1	82.1±0.1		95.9±2.5	9.0±0.5	*C*. *coli*
212262	Negative	NA	NA	NA	NA	NA	NA	Negative
212263	Negative	NA	78.8±0.0	81.5±0.0		5.4±0.2	71.1±1.8	*C*. *jejuni*
212264	Negative	NA	78.9±0.1	81.4±0.0		4.4±0.1	58.8±0.5	*C*. *jejuni*
212265	Negative	NA	78.3±0.0	81.5±0.0		6.8±0.2	92.2±0.7	*C*. *jejuni*
212266	Negative	NA	NA	NA	NA	NA	NA	Negative
212514	Positive	*C*. *jejuni*	78.5±0.0	81.9±0.1		5.5±0.1	91.8±0.6	*C*. *jejuni*
212515	Positive	*C*. *jejuni*	78.5±0.0	81.6±0.0		5.4±0.2	94.5±0.5	*C*. *jejuni*
212516	Negative	NA	NA	NA	NA	NA	NA	Negative
212517	Positive	*C*. *jejuni*	78.5±0.0	81.8±0.0		3.8±0.3	92.4±1.4	*C*. *jejuni*
212518	Positive	*C*. *jejuni*	78.5±0.0	81.8±0.0		7.6±0.1	90.3±2.0	*C*. *jejuni*
212519	Positive	*C*. *jejuni*	78.5±0.0	81.7±0.1		4.9±0.2	94.4±0.4	*C*. *jejuni*
212520	Positive	*C*. *jejuni*	78.5±0.0	81.7±0.1		6.6±0.1	91.9±1.8	*C*. *jejuni*
212521	Positive	*C*. *jejuni*	78.5±0.0	81.5±0.1		4.8±0.5	94.4±0.8	*C*. *jejuni*
212522	Positive	*C*. *jejuni*	78.7±0.0	80.9±0.1		5.9±0.7	89.8±4.1	*C*. *jejuni*
212523	Negative	NA	78.7±0.0	80.8±0.0		0.9±0.2	56.8±0.8	*C*. *jejuni*

^a^NA, Not applicable

## Discussion

This study describes a rapid and reliable mPCR-HRM technique for the differentiation of *C*. *jejuni* and *C*. *coli* isolates. The melting analysis of *Campylobacter* DNA, amplified with two sets of primers in a mixture containing a DNA intercalating dye (SYTO9) as a double-stranded DNA binding dye, allowed rapid detection of *Campylobacter* species *C*. *jejuni* and *C*. *coli*.

Although *C*. *jejuni* and *C*. *coli* are very close in their phenotypic and genotypic characteristics, the present study shows that the mPCR-HRM technique was able to differentiate between *C*. *jejuni* and *C*. *coli* using *hipO* and *asp* genes, respectively. Moreover, mPCR-HRM demonstrated some capability in the detection of minor variations within *C*. *jejuni* or *C*. *coli* species. Subtle differences in conventional and normalized curves within *C*. *jejuni* have been reported in the *flaA* gene [39]. The mPCR-HRM developed in this study, produced two distinct curve profiles for *C*. *jejuni* and *C*. *coli* species and could automatically differentiate the two species based on their GCPs without visual interpretation.

PCR-HRM curve analysis is a powerful and valuable tool to study the nucleotide diversity of amplicons between tested specimens. Results from this study indicate that mPCR-HRM has the potential to detect *Campylobacter* species and differentiate isolates based on their sequence variation of targeted gene. In HRM curve analysis, all samples are compared with a given arbitrary reference strain, and those that generate GCPs ≥ cut off point are considered similar to the reference strain while those that produce GCPs ≤ cut off point are considered as “variation”. Therefore samples genotyped as variation could well be different to the others. To study the possible difference between samples recognized as “variation” using a given arbitrary reference, another sample could be set up as reference genotype and samples can be reanalysed for their relationship with the reference. These analyses are conducted readily through the software provided with additional testing.

However, the PCR procedure could be susceptible to several factors such as quality and quantity of DNA template, annealing temperature between primers and DNA templates, self-annealing between PCR products and different copy numbers of the targeted genes. These factors may affect PCR results and subsequently HRM curve analysis. Therefore, optimization of the test including different quantities of DNA template and concentrations of PCR reagents were performed (data not shown).

Experience with HRM curve analysis in our laboratory has shown that the quantification of genomic DNA and use of an equal concentration of DNA for all tested specimens is useful for reliable amplification in PCR (unpublished data). However, equal concentrations of DNA may not be guaranteed in clinical samples, therefore whenever possible, adjustment of DNA to equal concentration would be beneficial for improving the consistency and reproducibility of HRM curve profiles to attain the least variation in curve shape [50]. The differences in amplicon sizes between *C*. *jejuni* and *C*. *coli* may also have contributed to the differentiation power of this technique due to the variations in nucleotide sequences and length.

In this study, comparable HRM curve profiles generated from three different sources, pure cultures of *Campylobacter* isolates, human faecal specimens and chicken carcase swab samples, demonstrated the consistency of the results.

Using equal quantity of template DNA, all faecal specimens containing *C*. *coli* produced two peaks in the conventional melt curve and all specimens containing *C*. *jejuni* generated only one peak (with or without shoulder peak) which were similar to the conventional melt curves produced from pure *Campylobacter* cultures. Therefore, when equal concentrations of template DNA were used, the quality of DNA did not have a significant effect on the consistency of the melting patterns. In addition, each sample has been tested in different runs/days and in triplicate. The Rotor Gene 1.7.27 software can automatically genotype the samples based on the provided cut off points and therefore, does not necessarily require skilful interpretation by the operator. This feature facilitates the application of the test in the routine diagnostic or research laboratories that the instrument is available. Similar tests are now being used in the routine diagnosis of *Mycoplasma gallisepticum* [51], infectious bursal disease virus [48], fowl adenovirus [35] and beak and feather disease virus [48] in our laboratory.

The melting profile of amplicons is based on the length and GC content of nucleotide sequence. As the amplicon begins to melt, DNA regions that contain more G/C compared to A/T, are more stable and do not melt immediately, instead, maintain their dsDNA configuration until the temperature is adequately high to cause it to melt. This phenomenon results in tow peaks in conventional melt curve. The first peak of *C*. *coli* and some of *C*. *jejuni* isolates is likely to be due to A-T rich region which melts at lower temperature and the second peak is likely to be due to melting G-C rich region at a higher temperature.

By using HRM in this study the risk of cross-contamination was reduced as PCR-HRM is a closed-tube technique. Other benefits of this technique include rapid testing and the opportunity to detect some intraspecies variations. All *C*. *jejuni* and *C*. *coli* isolates generated distinct conventional and normalized melt curves when compared with reference strains ATCC29428 and ATCC33559, respectively. The differences in HRM curves were a reflection of nucleotide sequence variation of the targeted gene in each isolate. Multiple sequence alignments of sequenced amplicons reaffirmed the HRM results (data not shown).

The HRM method may not replace sequence-based differential techniques and any new HRM curve profiles need to be confirmed by sequencing. However, once a new curve profile has been confirmed, it can be used in subsequent HRM analysis [37].

The melt curve profiles generated in this study were consistent and the melt curve profiles of each isolate obtained from different runs were similar with regard to the number, height, and temperature of the peaks. A DNA melting simulation program referred to as POLAND (Heinrich-Heine University in Dusseldorf, Germany, Institute of Biophysics [http://www.biophys.uni-duesseldorf.de/local/POLAND/poland.html]) [52] was also used to confirm whether mPCR-HRM curve analysis could potentially differentiate additional *C*. *jejuni* and *C*. *coli* strains that were unavailable in our laboratory. This program generates theoretical melting curve patterns according to the nucleotide sequence of a DNA fragment. The melting pattern of the *C*. *jejuni asp* gene and *C*. *coli hipO* gene (flanked by the primers used in this study) from selected number of *Campylobacter* strains were assessed in POLAND using sequences available in the GenBank database. The melt curve profiles predicted by POLAND from additional *Campylobacter* strains were in agreement with those generated in this study (data not shown). This also confirmed that the melting profile of *C*. *jejuni* and *C*. *coli* amplicons was as a result of DNA dissociation in mPCR-HRM.

The ability of mPCR-HRM curve analysis in detection and differentiation of *Campylobacter* species was further evaluated by testing additional nine human faecal samples from a different geographical location and 25 chicken carcase swab samples. All clinical specimens were genotyped as *C*. *jejuni* or *C*. *coli* except human faecal sample 11 and chicken swab samples 212252 and 212255 which contained both *C*. *jejuni* and *C*. *coli* species and generated different melt curves. The presence of more than one *Campylobacter* species in host animal [35] or human sample [36] has been reported. Although samples 5, 10, 50, 55 and 56 were genotyped as *C*. *jejuni*, their normalized curves were slightly different from ATCC29428 which was a reflection of slight variation in their nucleotide sequences (data not shown). Out of 25 chicken carcase swab samples, only eight were positive in culture while in mPCR-HRM, 22 samples were positive for *Campylobacter*. The higher sensitivity of mPCR-HRM compared with culture in detecting *Campylobacter* in swab samples could be due to the detection of genomic DNA by PCR from viable and nonviable bacteria. The clinical chicken samples that were genotyped as *C*. *jejuni* produced GCP values in a range of 56.8–96.3. Among all culture negative specimens, three samples (212258, 212264 and 212523) produced lower GCP values (<70) when compared with *C*. *jejuni* culture positive samples (Table 5). The lower GCP values in these samples could be due to lower number of organism present in the sample. Comparison of equal volumes of amplicons on agarose gel showed relatively lower concentration of amplicon in these three specimens (data not shown). Samples with variable concentrations of starting DNA template produce different amount of fluorescence in HRM [50]. This along with lower concentration of target sequence in the amplicon could have contributed to the lower GCP values. However, by comparing the peak melting points and normalized melt curves (Fig 5b), similar *Campylobacter* species were genotyped within the same cluster.

The mPCR-HRM developed in this study represents a relatively simple method for discrimination between *C*.*jejuni* and *C*. *coli* species and also has the potential to detect differences between isolates. This could be most useful for screening of clinical specimens as these samples may hypothetically contain *C*. *coli*, *C*. *jejuni*, both species or an unknown field strain. This however should be readily detectable given that the melting curve profile of *C*. *coli* and *C*. *jejuni* are distinct and already characterized. Where “variation” is detected in clinical specimens from such specimens, the presence of the isolate is best to be confirmed using additional tests such as PCR followed by nucleotide sequencing of the amplicons.

The PFGE still could be considered as a method which has a high discriminatory power in differentiating *Campylobacter* species. However, it is a time consuming method and requires standard protocol and skilled technician and relatedness among samples are used as a guide not true phylogenetic measure [53].

The newly developed mPCR-HRM is faster and more cost-effective than the other laboratory methods such as Taqman PCR for differentiating these species [54, 55]. Nucleotide sequencing is believed to be the gold standard for detection of sequence variations, however, in a meta analysis, the HRM method was considered as one of the preferred methods in detection of sequence variation among current available techniques and a high sensitive modality when compared with DNA sequencing [56]. The significant advantage of HRM curve analysis relies on differentiation of isolates based on variation of their nucleotide sequences without requiring nucleotide sequencing [38, 57]. The multiplex-PCR HRM curve analysis described in this study can differentiate *C*. *jejuni* and *C*. *coli* without requiring enrichment or isolating bacteria prior to testing as well as discriminating the intraspecies within each species. In addition, mPCR-HRM curve analysis proved to be rapid, inexpensive, requires minimum requirement for interpretation, and is amenable to automation for screening of a large number of specimens.

## Supporting Information

S1 Fig Agarose gel electrophoresis of mPCR products for *C*. *coli* (500 bp) and *C*. *jejuni* (735 bp) strains/isolates using *asp* and *hipO* genes, respectively. MW, molecular weight marker (PCR Marker, Sigma).
(TIF)

S2 Fig (a) Conventional melt curve and (b) normalized HRM curve analysis of mPCR amplicons of the 2 *C*. *jejuni* specimens ATCC29428 and BAL172643 (TIF)

S3 Fig The genotypes identified by HRM curve analysis and phylogenetic relationship of (a) *C*. *jejuni* isolates based on amplicon sequence of *hipO* gene, (b) *C*. *coli* isolates based on amplicon sequence of *asp* gene. (TIF)

S4 Fig The phylogenetic relationship of *Campylobacter* isolates from clinical samples based on nucleotide sequence of *asp* gene (*C*. *coli*) and *hipO* gene (*C*. *jejuni*). Sample 11 contained *C*. *coli* (11C) and *C*. *jejuni* (11J). (TIF)

S1 Table Percentage of sequence identity and diversity between nine *C*. *coli* isolates. (DOCX)

S2 Table Percentage of sequence identity and diversity between 17 *C*. *jejuni* isolates. (DOCX)
