# Supplementary figures and images for: Differentiation of Campylobacter jejuni and Campylobacter coli Using Multiplex-PCR and High Resolution Melt Curve Analysis
Source: PLoS One. 2015 Sep 22;10(9):e0138808. doi: 10.1371/journal.pone.0138808 (PMC4578860; doi:10.1371/journal.pone.0138808)

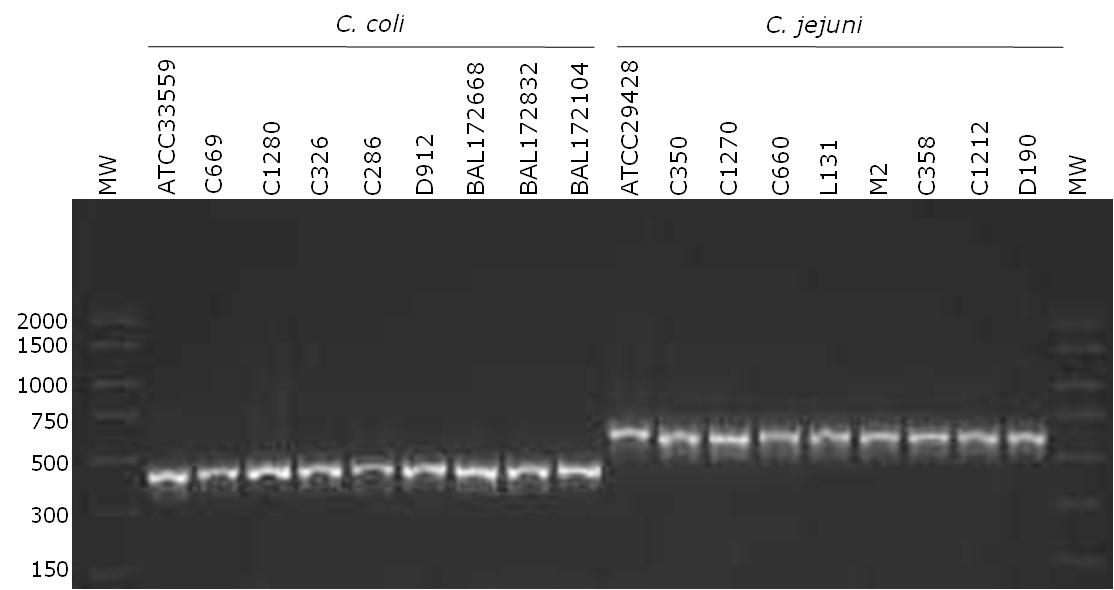

Supplement: S1 Fig — MW, molecular weight marker (PCR Marker, Sigma). (TIF) [file pone.0138808.s001.tif]

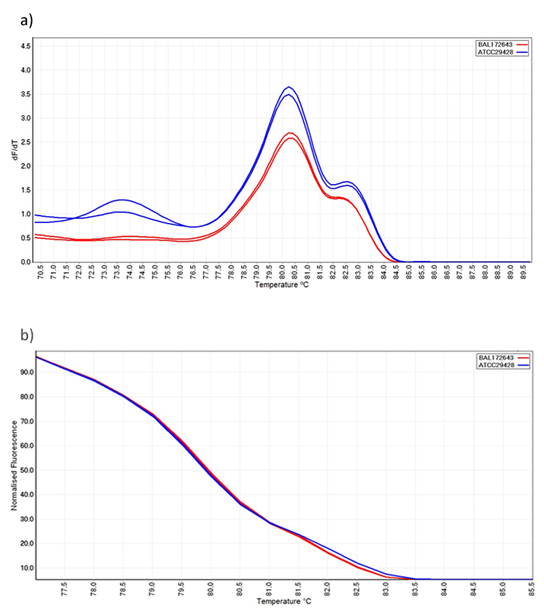

Supplement: S2 Fig — (TIF) [file pone.0138808.s002.tif]

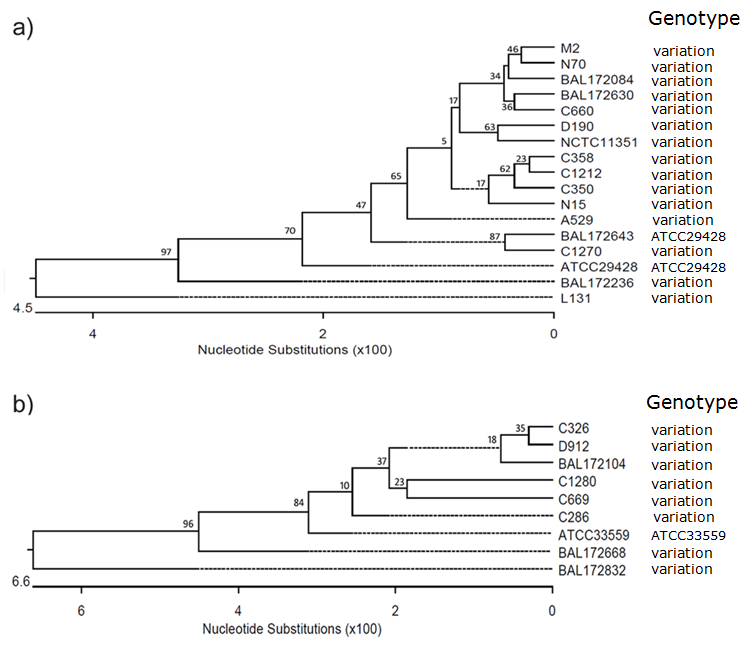

Supplement: S3 Fig — (TIF) [file pone.0138808.s003.tif]

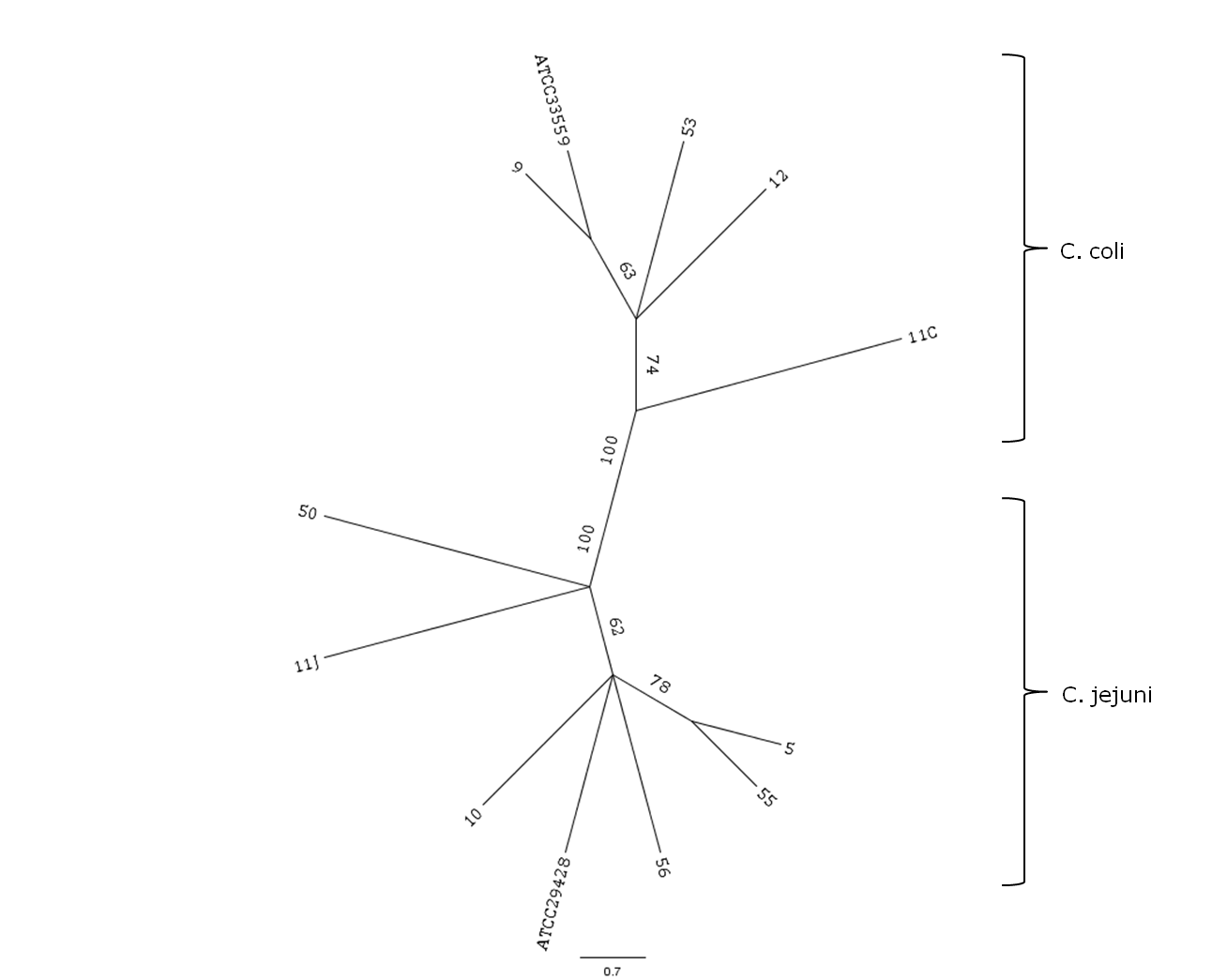

Supplement: S4 Fig — (TIF) [file pone.0138808.s004.tif]
