# Supplementary material for: Differentiation of Campylobacter jejuni and Campylobacter coli Using Multiplex-PCR and High Resolution Melt Curve Analysis
Source: PLoS One. 2015 Sep 22;10(9):e0138808. doi: 10.1371/journal.pone.0138808 (PMC4578860; doi:10.1371/journal.pone.0138808)
Supplement: S1 Table — (DOCX) [file pone.0138808.s005.docx]

**Table S1.** Percentage of sequence identity and diversity between nine *C. coli* isolates.

|  | Sequence identity | | | | | | | | |
| --- | --- | --- | --- | --- | --- | --- | --- | --- | --- |
| Isolate | ATCC33559 | BAL172104 | BAL172668 | BAL172832 | C1280 | C286 | C326 | C669 | D912 |
| ATCC33559 | - | 97.4 | 95.9 | 96.4 | 95.2 | 98.8 | 97.0 | 97.2 | 98.0 |
| BAL172104 | 2.9 | - | 94.8 | 97.4 | 96.6 | 98.6 | 98.8 | 97.8 | 98.4 |
| BAL172668 | 3.3 | 3.8 | - | 96.5 | 94.6 | 95.7 | 95.5 | 93.5 | 94.6 |
| BAL172832 | 7.3 | 8.2 | 2.1 | - | 93.9 | 98.8 | 97.4 | 96.0 | 96.4 |
| C1280 | 3.7 | 2.4 | 4.3 | 8.9 | - | 95.6 | 95.8 | 95.6 | 95.6 |
| C286 | 1.4 | 1.4 | 2.7 | 6.6 | 3.5 | - | 98.2 | 97.6 | 97.8 |
| C326 | 3.3 | 1.2 | 4.0 | 8.0 | 3.3 | 1.8 | - | 96.6 | 99.2 |
| C669 | 3.5 | 2.7 | 5.0 | 9.1 | 3.7 | 2.9 | 3.9 | - | 96.8 |
| D912 | 2.6 | 1.4 | 3.8 | 8.4 | 3.0 | 2.0 | 0.6 | 3.3 | - |
|  | Sequence diversity | | | | | | | | |
