# Supplementary material for: Differentiation of Campylobacter jejuni and Campylobacter coli Using Multiplex-PCR and High Resolution Melt Curve Analysis
Source: PLoS One. 2015 Sep 22;10(9):e0138808. doi: 10.1371/journal.pone.0138808 (PMC4578860; doi:10.1371/journal.pone.0138808)
Supplement: S2 Table — (DOCX) [file pone.0138808.s006.docx]

**Table S2.** Percentage of sequence identity and diversity between 17 *C. jejuni* isolates.

|  | Sequence identity | | | | | | | | | | | | | | | | | |
| --- | --- | --- | --- | --- | --- | --- | --- | --- | --- | --- | --- | --- | --- | --- | --- | --- | --- | --- |
| Isolate | ATCC29428 | BAL172084 | BAL172236 | BAL172630 | | BAL172643 | C1212 | C1270 | C350 | C358 | C660 | D190 | L131 | M2 | N15 | N70 | NCTC11351 | A529 |
| ATCC29428 | - | 95.3 | 96.3 | 96.3 | | 98.1 | 96.8 | 97.0 | 96.6 | 96.8 | 96.8 | 96.7 | 97.3 | 97.1 | 97.1 | 97.0 | 96.7 | 97.8 |
| BAL172084 | 2.5 | - | 97.6 | 98.6 | | 98.9 | 97.7 | 98.0 | 98.2 | 98.2 | 98.6 | 97.9 | 96.2 | 98.2 | 98.3 | 98.6 | 97.7 | 97.9 |
| BAL172236 | 1.8 | 2.5 | - | 96.9 | | 97.7 | 97.0 | 97.7 | 97.0 | 97.0 | 96.9 | 96.7 | 99.0 | 96.6 | 97.4 | 96.9 | 96.6 | 97.9 |
| BAL172630 | 2.4 | 0.9 | 2.9 | - | | 98.4 | 98.6 | 98.1 | 98.8 | 99.0 | 99.3 | 98.6 | 97.0 | 98.6 | 99.0 | 99.2 | 97.9 | 98.8 |
| BAL172643 | 1.2 | 1.0 | 2.0 | 1.5 | | - | 98.0 | 99.4 | 98.4 | 98.4 | 98.3 | 97.9 | 97.0 | 98.1 | 98.6 | 98.3 | 97.4 | 98.7 |
| C1212 | 2.5 | 1.7 | 2.8 | 1.4 | | 1.7 | - | 97.7 | 99.2 | 99.6 | 98.8 | 98.4 | 97.8 | 98.4 | 99.1 | 98.6 | 98.1 | 98.5 |
| C1270 | 2.4 | 1.3 | 1.9 | 1.8 | | 0.1 | 2.4 | - | 97.7 | 98.1 | 98.0 | 97.6 | 97.4 | 98.1 | 98.0 | 98.1 | 97.4 | 98.4 |
| C350 | 2.8 | 1.3 | 2.8 | 1.2 | | 1.3 | 0.8 | 2.3 | - | 99.5 | 98.6 | 97.8 | 97.4 | 98.0 | 98.6 | 98.1 | 97.6 | 98.2 |
| C358 | 2.5 | 1.3 | 2.8 | 1.0 | | 1.3 | 0.4 | 1.9 | 0.5 | - | 98.9 | 98.2 | 97.7 | 98.6 | 98.9 | 98.8 | 98.1 | 98.6 |
| C660 | 2.5 | 0.9 | 2.9 | 0.7 | | 1.5 | 1.2 | 2.1 | 1.4 | 1.1 | - | 98.6 | 97.7 | 99.2 | 98.9 | 99.3 | 98.2 | 98.9 |
| D190 | 2.5 | 1.6 | 3.1 | 1.4 | | 1.9 | 1.5 | 2.4 | 2.1 | 1.7 | 1.4 | - | 97.1 | 98.2 | 98.6 | 98.8 | 99.2 | 98.2 |
| L131 | 5.0 | 3.1 | 0.4 | 5.6 | | 1.9 | 5.5 | 5.2 | 5.8 | 5.7 | 5.7 | 6.0 | - | 97.7 | 97.8 | 97.4 | 97.3 | 98.6 |
| M2 | 2.8 | 0.7 | 2.5 | 0.8 | | 1.0 | 1.8 | 2.2 | 1.7 | 1.0 | 1.0 | 1.8 | 5.1 | - | 98.2 | 99.2 | 98.4 | 98.8 |
| N15 | 2.2 | 1.1 | 2.3 | 1.0 | | 1.2 | 1.0 | 2.1 | 1.2 | 1.1 | 1.1 | 1.2 | 5.2 | 1.4 | - | 98.6 | 97.8 | 98.6 |
| N70 | 2.9 | 0.9 | 2.9 | 0.8 | | 1.5 | 1.5 | 2.5 | 1.6 | 0.8 | 0.8 | 1.7 | 5.2 | 0.5 | 1.1 | - | 98.2 | 98.8 |
| NCTC11351 | 3.1 | 1.6 | 2.8 | 1.9 | | 1.6 | 2.4 | 2.9 | 2.2 | 2.1 | 2.1 | 1.0 | 5.2 | 1.2 | 1.9 | 1.7 | - | 98.4 |
| A529 | 1.5 | 1.6 | 1.9 | 1.2 | 1.0 | 1.5 | 1.6 | 1.8 | 1.1 | 1.1 | 1.7 | 4.7 | 1.4 | 1.4 | 1.4 | | 2.2 | - |
|  | sequence diversity | | | | | | | | | | | | | | | | | |
